# Viime: Visualization and Integration of Metabolomics Experiments

**DOI:** 10.21105/joss.02410

**Published:** 2020-10-18

**Authors:** Roni Choudhury, Jon Beezley, Brandon Davis, Jared Tomeck, Samuel Gratzl, Lilian Golzarri-Arroyo, Jun Wan, Daniel Raftery, Jeff Baumes, Thomas M. O’Connell

**Affiliations:** 1 Kitware Inc.; 2 Department of Epidemiology and Biostatistics, Indiana University School of Public Health; 3 Department of Medical and Molecular Genetics, Indiana University School of Medicine; 4 Center for Computational Biology and Bioinformatics, Indiana University School of Medicine; 5 Department of BioHealth Informatics, Indiana University School of Informatics and Computing; 6 Department of Anesthesiology and Pain Medicine, University of Washington; 7 Department of Otolaryngology–Head and Neck Surgery, Indiana University School of Medicine

## Abstract

Metabolomics involves the comprehensive measurement of metabolites from a biological system. The resulting metabolite profiles are influenced by genetics, lifestyle, biological stresses, disease, diet and the environment and therefore provides a more holistic biological readout of the pathological condition of the organism (Beger et al., 2016; Wishart, 2016). The challenge for metabolomics is that no single analytical platform can provide a truly comprehensive coverage of the metabolome. The most commonly used platforms are based on mass-spectrometry (MS) and nuclear magnetic resonance (NMR). Investigators are increasingly using both methods to increase the metabolite coverage. The challenge for this type of multi-platform approach is that the data structure may be very different in these two platforms. For example, NMR data may be reported as a list of spectral features, e.g., bins or peaks with arbitrary intensity units or more directly with named metabolites reported in concentration units ranging from micromolar to millimolar. Some MS approaches can also provide data in the form of identified metabolite concentrations, but given the superior sensitivity of MS, the concentrations can be several orders of magnitude lower than for NMR. Other MS approaches yield data in the form of arbitrary response units where the dynamic range can be more than 6 orders of magnitude. Importantly, the variability and reproducibility of the data may differ across platforms. Given the diversity of data structures (i.e., magnitude and dynamic range) integrating the data from multiple platforms can be challenging. This often leads investigators to analyze the datasets separately, which prevents the observation of potentially interesting relationships and correlations between metabolites detected on different platforms. Viime (VIsualization and Integration of Metabolomics Experiments) is an open-source, web-based application designed to integrate metabolomics data from multiple platforms. The workflow of Viime for data integration and visualization is shown in Figure 1.

## User Interface Features and Architecture

### Data Upload

Data upload can be a cumbersome step in many data analysis packages. Often the data must be provided in a specified format in order to be properly read and the details of the requisite format are not always clear. To facilitate the easy import of data, we have designed an interactive drag and drop data upload interface which currently accepts .xlsx and .csv files.

**Figure 1:**
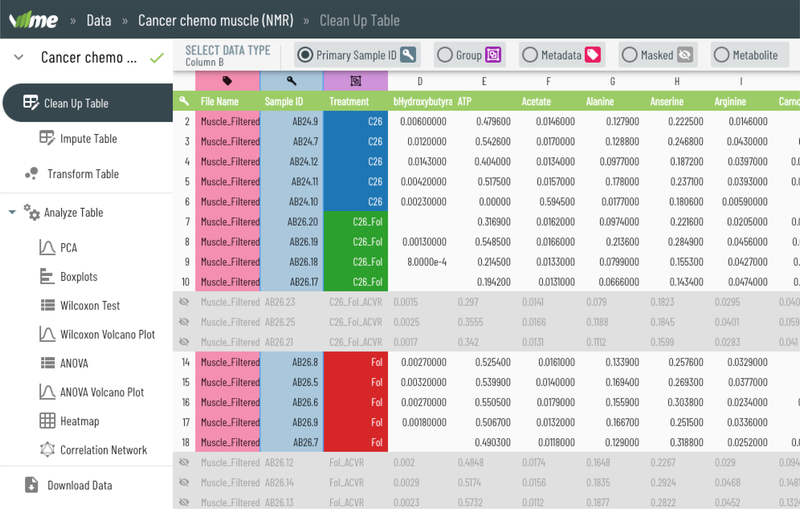
The data ingestion view.

The UI begins by presenting the user with an upload screen, which reports whether any errors were encountered in the file. The user then is able to correct any errors, designating any column as the primary ID, masked/hidden, a factor, the group, or a metabolite concentration column (see Figure 1). The table view and its associated server support have been designed to support tables that scale to hundreds of rows and thousands of columns, enabling support for a wide range of experimental data sizes.

**Figure 2:**
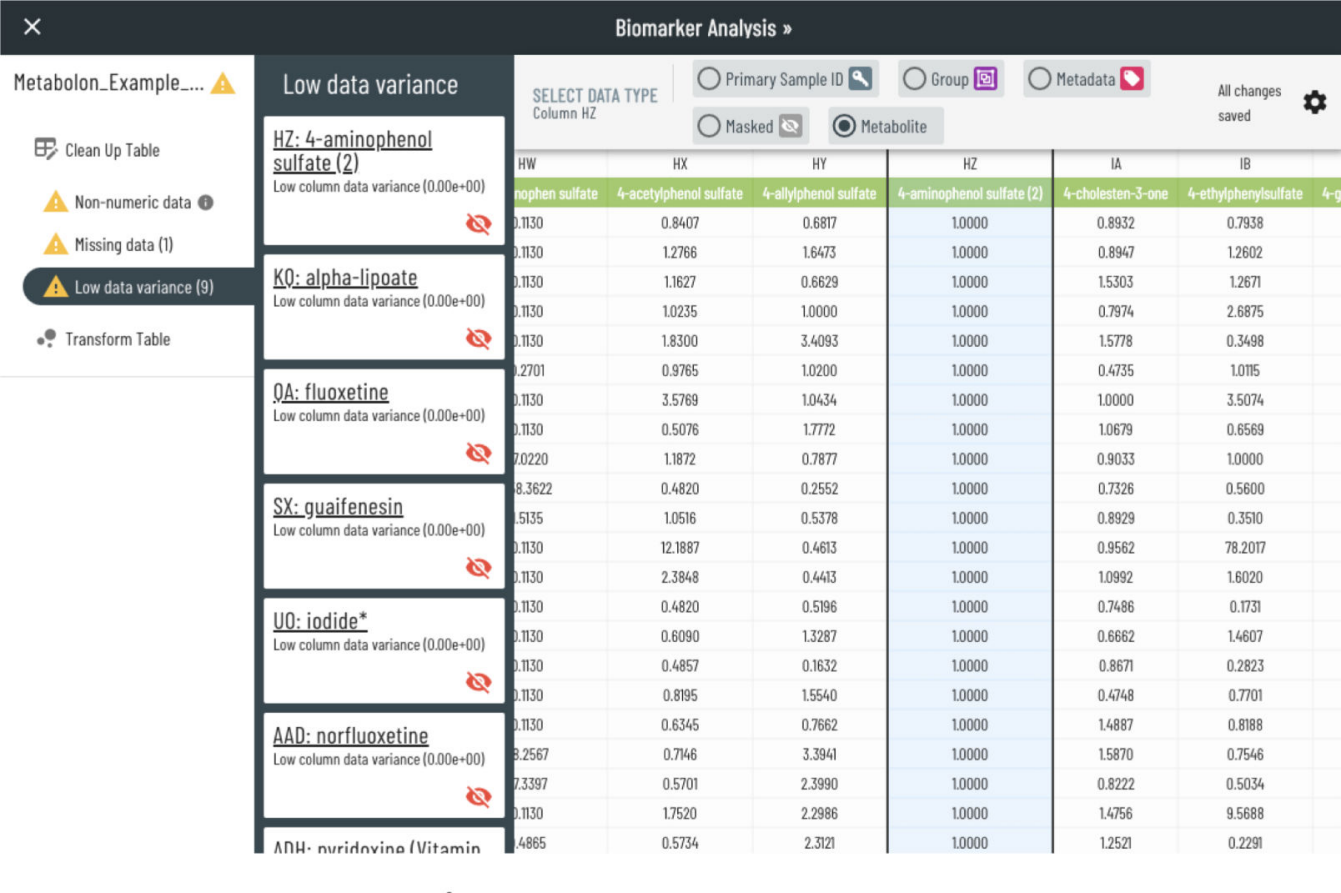
The ingestion error and warning panel.

Any errors encountered during parsing are prompted for correction. Errors that are detected include levels of missing data that exceed a default threshold within a group or across all samples, non-numeric data in concentration data, the lack of a primary ID, and non-uniqueness of the primary ID. The UI guides the user through each error and warning until the data is ready for analysis. As seen in Figure 2, a low-variance metabolite is being flagged for possible omission from analysis.

### Data Imputation

Once errors are corrected, data imputation is automatically performed. For metabolites with missing values, the type of missingness is classified as missing completely at random (MCAR) or missing not at random (MNAR). For each type, an imputation mode is automatically performed but the options may be adjusted to apply different algorithms, including random forest, K neareset neighbors, mean, or median imputation modes for completely at random missingness (MCAR) and zero or half-minimum imputation for not-at-random missingness (MNAR).

### Dataset Details

**Figure 4:**
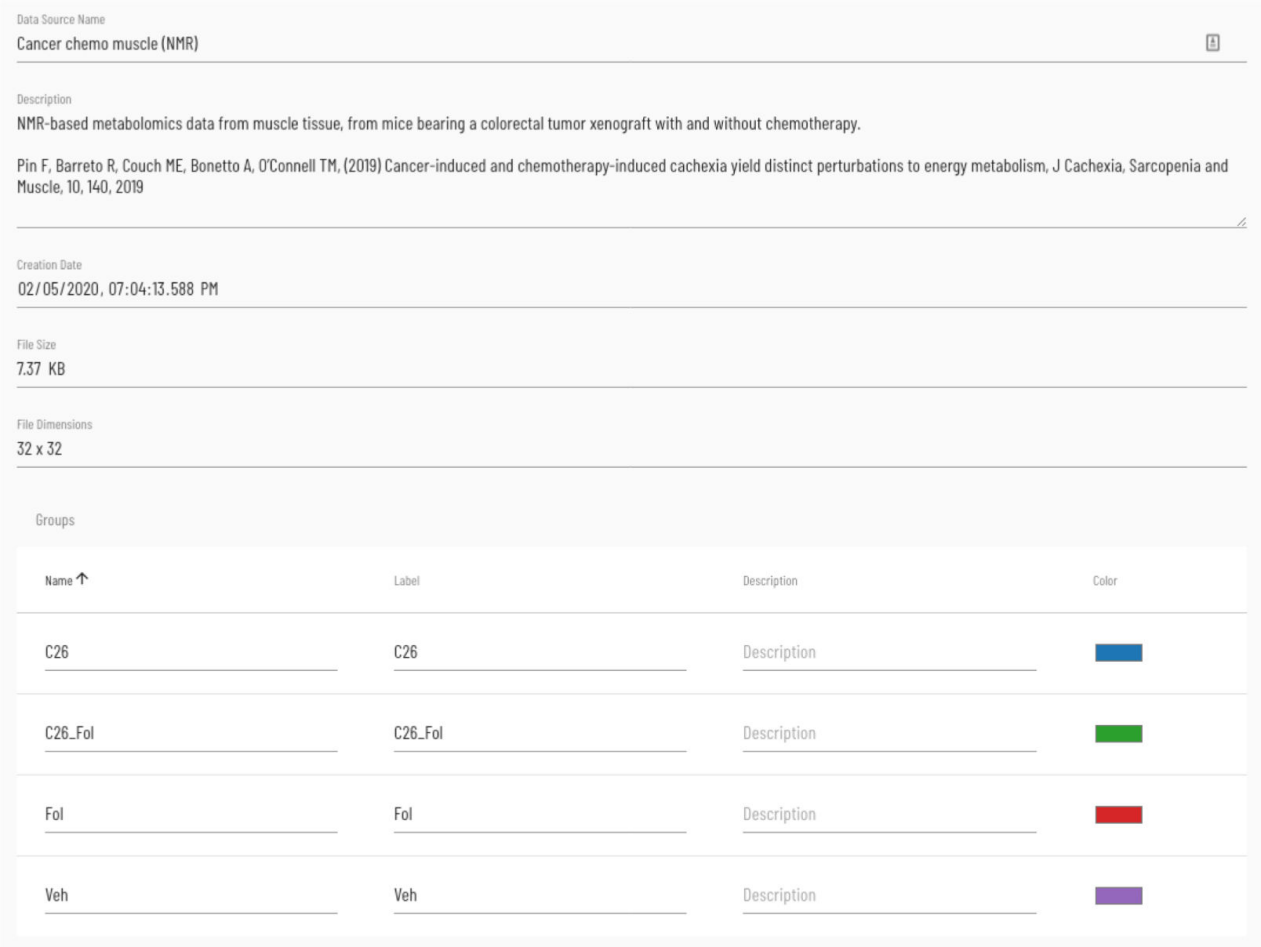
Dataset details page.

Viime includes a dataset details page which includes size, creation time, and enables the user to update the name and description for each dataset (see Figure 4). It is also a central location for assigning colors and descriptions to groups, and keeping track of provenance for merged datasets.

A download page enables users to export their cleaned and processed dataset, or download the currently selected metabolite list.

### Data Treatment

The most critical step in the process of integrating multiple datasets is setting the optimal data treatment parameters for the individual datasets. The first step in this process is data normalization. In this step, the measurement values of each sample are made consistent with the other samples in the dataset. This can be accomplished by normalizing values of each sample to that of a reference sample. In this process, the sum of all metabolite values for the reference sample is determine and this value is then used to provide a normalization factor for the other samples based on their metabolite sums. Similarly, the sum of all values for each sample can be scaled to a set value. The default value in Viime is 100. Other options include normalization based on a column containing sample weights or volumes. The next step is data transformation. Often times, data is transformed to bring the distribution closer to normality and to compress the dynamic range. The options in this step are Log10, Log2, square root and cube root.

The final step is scaling. This also addresses the issue of large dynamic range by scaling the variance of the data. The options here include autoscaling, Pareto scaling, range scaling, vast scaling and level scaling.

A very important feature of the whole data treatment process is the interactive use of principal component analysis (PCA) to examine the similarity and dissimilarity of individual groups in the dataset for different data treatment options. Viime provides an interactive PCA score plot, showing how the selection of each treatment option affects the separation of the individual groups in the data. In this way, a user can quickly examine a number of different treatments to better understand their data. A loadings plot shows how each treatment option affects the contributions of the metabolites to the separations. Often data with no transformation or scaling may be dominated by only a few of the very high concentration metabolites. In those cases, some separation of the groups may be present, but are the result of looking at only those metabolites. Autoscaling is often a default selection in some metabolomics data analysis packages, but this runs the risk of increasing the noise in the data. This is characterized by a loadings plot where all of the metabolites display large loading values which is typically not a biologically plausible condition (Berg, Hoefsloot, Westerhuis, Smilde, & Werf, 2006).

### Data Analysis and Visualization

Viime supports several downstream analyses and visualizations. Univariate analyses using the Wilcoxon rank sum test and multivariate ANOVA can be carried out on data with two or more groups, respectively. For the ANOVA, a post-hoc Tukey test is automatically applied so that p-values for each of the inter-group comparisons are calculated for all metabolites. Metabolites that are significantly different in each of the intergroup comparisons can be selected for further analysis with a check box at the top of each column. This enables very large datasets with potentially hundreds of metabolites to be easily reduced to datasets containing only significantly altered metabolites.

#### Volcano plots

To simultaneously visualize the magnitude of the change in a metabolite along with the statistical significance of that change, Viime offers an interactive volcano plot option. As shown in Figure 8, the horizontal axis displays the Log2 Fold change while the vertical axis displays the −log10 of the p-value. This type of plot is useful when making two-group comparisons; the specific group pairs can be selected from the Group Combination menu. The minimum fold change and p-values can be interactively adjusted to highlight larger or smaller metabolite changes.

#### Heatmaps

**Figure 6:**
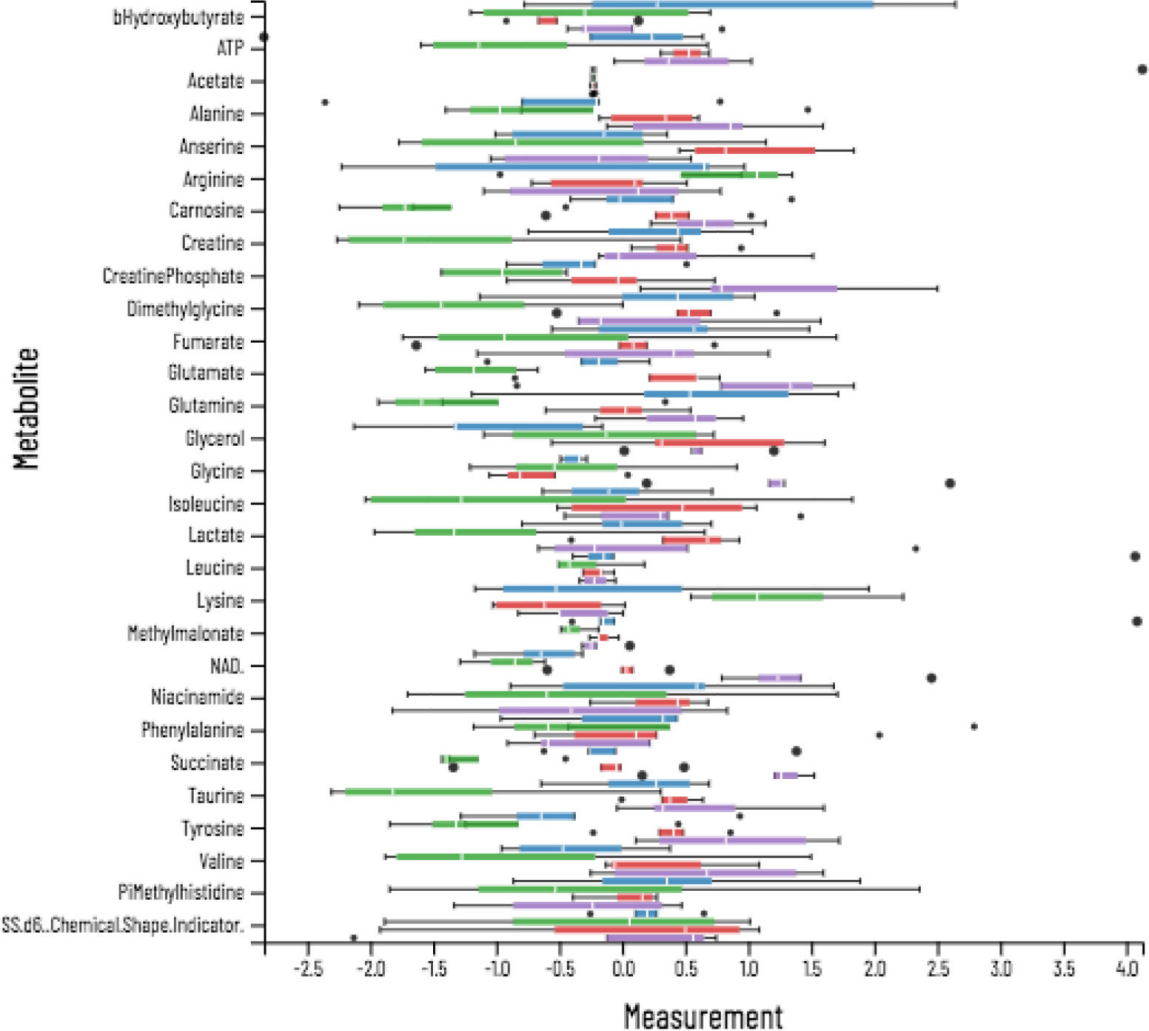
Boxplots of each metabolite, colored and separated by experimental group.

Heatmaps of the data can be generated to help visualize metabolites changes (Figure 6). The metabolite filter option on the Heatmaps page allows the option to include all metabolites in the heatmap versus only the significant metabolites. When the dataset is comprised of data from multiple platforms, the metabolite filter option also enables the selection of data from any of the separate platforms. The Sample Filter option allows only specific groups of samples to be included in the heatmap. The metabolite color option changes the color along with the vertical axis related to the metabolites. The options include coloring based on significance or based on data source. Hierarchical clustering analysis is carried out on both the samples and metabolites to help cluster the most similar sample and metabolite patterns. Both of these options can be toggled on or off if it would be beneficial to maintain the order of the samples and/or metabolites in the heatmap.

#### Network Correlation Diagrams

An interactive spring-embedded metabolite network correlation diagram can be generated for the data. The plot contains nodes for all of the metabolites connected by edges when the correlation between metabolite pairs is sufficiently high. The Methods options enable the correlations to be based on Pearson, Kendall Tau or Spearman rank correlations. The Node Filter and Node Color options enables the nodes to be selected or colored based on the data source or significance. The advanced options enable all metabolite nodes or edges to be labeled. The minimum correlation used for visualization can be interactively adjusted. Using the left mouse button the map can be moved and using the wheel, the map can be expanded. To help clean up and interrogate the data, individual metabolites can be selected, moved and pinned in the map. This enables a cleaner visualization of selected metabolite groups. Hovering over nodes or edges brings up the metabolite identification information and the strength of the correlations respectively.

Viime also includes a fully interactive heatmap with row and column dendrograms (see Figure 6). Selected metabolites are highlighted in orange on the left. Sample groups are colored along the bottom to provide additional context.

**Figure 7:**
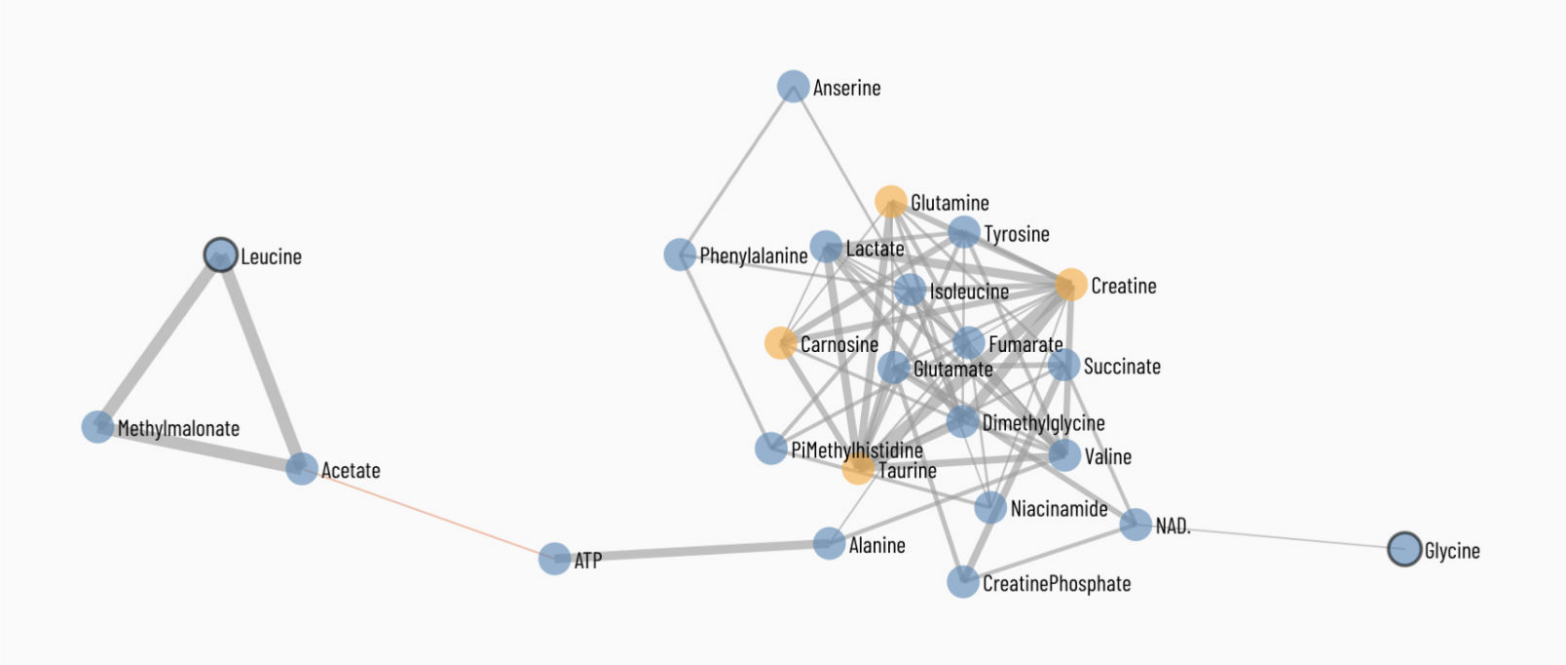
Correlation network diagram.

Unique to Viime is a metabolite correlation network diagram (see Figure 7). The color in the diagram represents whether the metabolite was significantly different across groups (orange) or not (blue). Metabolites are linked if the correlation coefficient between them exceeds a configurable value. Negative correlations are in red, while positive correlations are in gray. The width of the link encodes the strength of the correlation.

**Figure 8:**
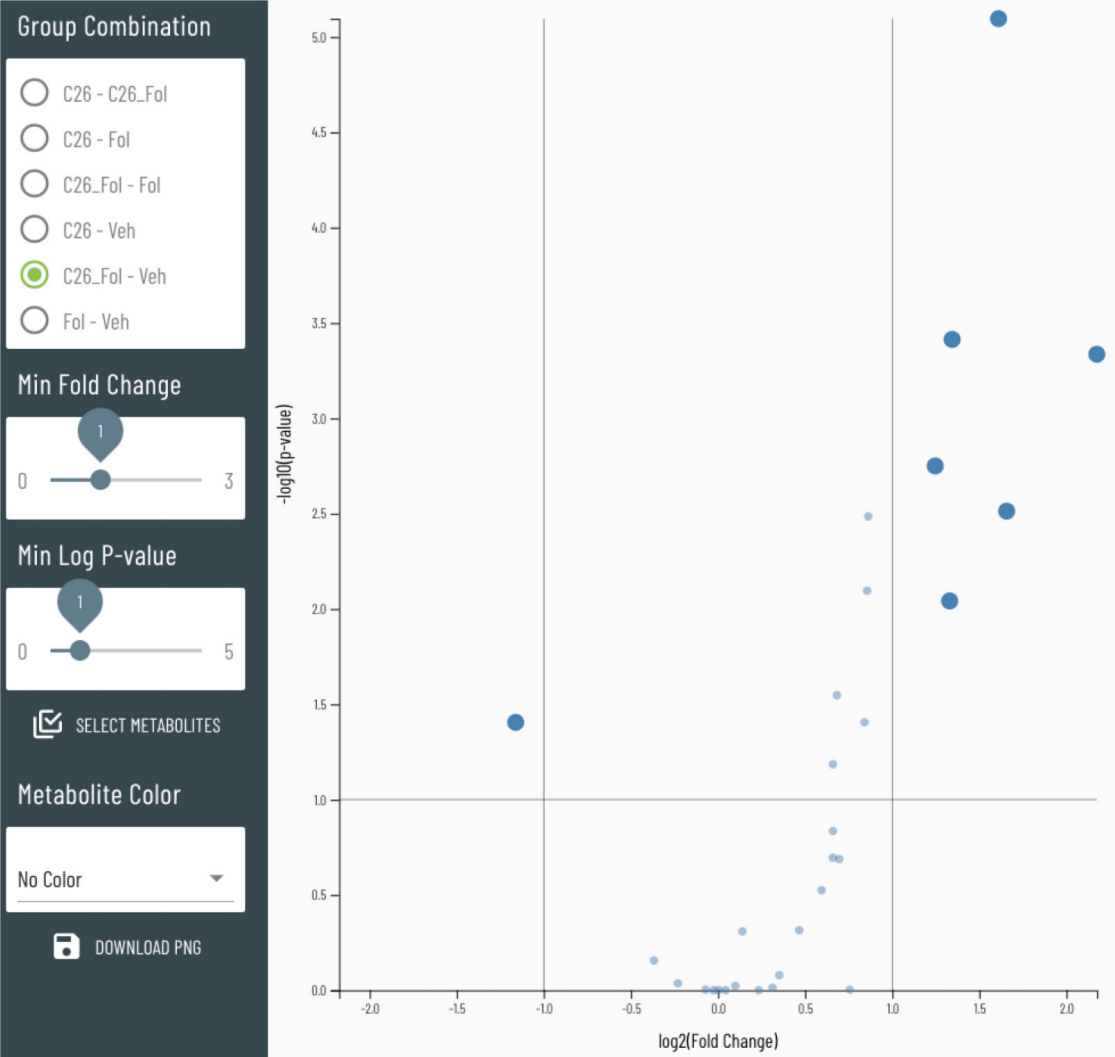
Volcano plot with interactive controls.

Volcano plots (see Figure 8) were added to the software to highlight the metabolites that meet a specified threshold for fold change and significance (p-value). For datasets with only two groups, the data from the Wilcoxon analysis is plotted. Interactive threshold adjustments for both fold change and p-value enable a simplified view. For datasets with more than two groups, the data from an ANOVA analysis is used and has options to plot data from selected groups. Options include selecting the group combination to analyze, the minimum fold change to highlight, and the minimum p-value to highlight. The thresholds are live controls which provide immediate feedback showing which metabolites meet the criteria. Once the proper thresholds are set, the user may download the resulting plot image, and also can save and download the metabolites that fall into above the thresholds. When the significant metabolites are selected, the user may move to any other plot to see those same metabolites highlighted in a different context, such as the heatmap view or correlation network.

### Data Integration

Viime supports multiple approaches for combining multiple data sources into a joint analysis. From the data upload page, the user may initiate a dataset merge, selecting the datasets to merge along with the algorithm to perform the integration.

**Figure 9:**
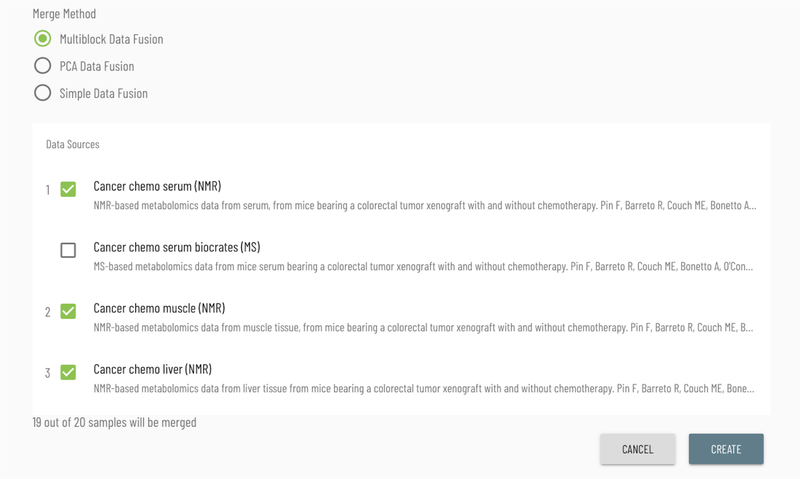
The interface for selecting the data and algorithm for integration.

Supported algorithms are simple column concatenation, PCA data fusion (concatenating the normalized scores of PCAs applied to each data set, keeping all variables and avoiding any loss of information, and leaving only common major effects (Spiteri et al., 2016)), and multi-block PCA fusion (by normalizing each of the individual data sets so that their first principal component has the same length (as measured by the first singular value of each data table) and then combining these data tables to a grand table. (Abdi, Williams, & Valentin, 2013)). After choosing an algorithm and two or more datasets, the interface indicates how many of the samples will match after the merging process. When the integration algorithm completes, the new integrated dataset appears in the list of data for the user to perform analyses (see Figure 9).

## Backend Processing

Viime’s processing backend is implemented as a RESTful API using the Flask web framework. Data persistence is provided through normalized CSV files stored on a filesystem and associated data in a SQLite database through SQLAlchemy’s ORM. Files stored internally are linked with rows in the database using custom fields provided by File Depot (“DEPOT,” n.d.). The backend leverages Pandas for raw file parsing and normalization. Data processing is done by a combination of Scikit-learn for common statistical algorithms and R packages for specialized algorithms. The R-python integration is provided by a secondary REST service exposed internally via OpenCPU.

Upon uploading a new dataset from an Excel or raw CSV file, the server begins by constructing a Pandas dataframe. Any parsing errors due to malformed files immediately result in an error response from the server. The Pandas object is used to populate a new row in the primary data table with associated metadata and processing defaults. Every row and column from the parsed dataset is also added to related tables including header information, detected data type, and an initial table structure determining properties such as which rows and columns contain metadata, group information, or raw metabolite values.

The cleanup phase of the workflow allows users to override the initial table structure for example by marking specific columns as metadata or by “masking” rows so they are ignored in the processing steps. Each time the user makes a change to the table structure the dataset is processed by a validation function that determines whether the dataset is ready for processing. This validation checks many properties of the dataset including that all metabolite values are numeric and metabolite names are unique. In addition, the validation will warn the user of likely problems such as too many missing or “not a number” values within a metabolite or group or columns containing an excessively low variance.

Once validated, the original dataset is broken down into three tables, one containing the raw metabolite measurements and two containing metadata about each row and column in the measurement table. The measurement table is then processed through imputation which fills in missing data using a series of user-configurable algorithms. A function defines which metabolites have missing data according to the Missing Not at Random (MNAR) or Missing Completely At Random (MCAR) models, depending on the percentage of missing values per group per metabolite. For each type of missingness the user can choose different imputation methods; MNAR allows users to impute using the Zero or Half Minimum strategies while MCAR allows imputation via Random Forest, K-Nearest Neighbor, Mean, or Median. Most of the imputation methods were implemented in R, while Random Forest and K-Nearest Neighbor were implemented with the R packages missForest and impute, respectively.

Before statistical analysis is performed, the imputed dataset is passed through a series of optional, user-configurable preprocessing steps including normalization (min max, sum, reference Sample, weight/volume), transformation (log base 10, log base 2, square root, cube root), and scaling (autoscaling, Pareto Scaling, range scaling, vast scaling, level scaling). All preprocessing functions were programmed in R. After preprocessing, the dataset is ready for input into the analysis methods.

## Related Work

New analytical approaches to effectively measure more and more of the metabolome are continually being developed. The data produced from these different approaches requires different handling in order to transform the data into useful biological information. Recently, Spicer et al. (Spicer, Salek, Moreno, Cañueto, & Steinbeck, 2017) reviewed the most popular freely-available software tools for metabolomics analysis. Based on their intended functionality the tools were classified into the following five groups: pre-processing, annotation, post-processing, statistical analysis, and workflows. Pre-processing and annotation tools are often very specific to the type, make, and model of the analytical instrument used to collect the data and therefore require specific tools. Once these steps are carried out, more general workflow tools can be used to complete the analysis. The intent of Viime package is to pick up the workflow after preprocessing and annotation and go all the way through statistical analysis and visualization. Note that annotation is not required and data can be analyzed that is unannotated or incompletely annotated.

Spicer et al., briefly described seven popular metabolomics workflow packages that met a threshold of at least 50 citations on Web of Science (as of August 2016) or were reported in a recent survey of the Metabolomics Society. The packages MZmine (Pluskal, Castillo, Villar-Briones, & Orešič, 2010) and MAIT (Fernández-Albert, Llorach, Andrés-Lacueva, & Perera, 2014) specifically focus on the analysis of mass-spectrometry data. The MAVEN package (Clasquin, Melamud, & Rabinowitz, 2012; Melamud, Vastag, & Rabinowitz, 2010) is focused on isotope tracer studies. The Workflow4Metabolomics (Giacomoni et al., 2014) and Galaxy-M (Davidson, Weber, Liu, Sharma-Oates, & Viant, 2016) packages are built upon the Galaxy web-based platform and are composed of various modules and workflows. Among the most well-known metabolomics workflow tools are MetaboAnalyst (Chong & Xia, 2020) and XCMS Online (Tautenhahn, Patti, Rinehart, & Siuzdak, 2012). These are both workflow tools which include MS spectral processing and have statistical analyses and visualization tools that are generally similar to Viime.

An exhaustive feature comparison with these other platforms is beyond the scope of this paper, but a major distinguishing feature of Viime is its emphasis on ease of use and interactivity. Only XCMS and MetaboAnalyst are simple, readily accessible web applications that require no existing package (e.g., R), downloads or connection to the Galaxy platform. The unique user interactivity in Viime starts with the ability to simply drag and drop CSV or Excel files and interactively assign the sample identifiers, comparison groups, metadata, and metabolites. Dynamic visualization of the PCA scores and loadings plots with different types of data (e.g., NMR, LC-MS, and GC-MS) and data treatments (e.g., normalization, scaling and transformation) aids in selecting the optimal data treatment. Viime also enables integration between different data modalities, offering simple (i.e., concatenative), mid-level, and multi-block data fusion approaches. The resulting fused datasets offer expanded metabolome coverage, enabling an analysis of the correlated behavior of metabolites detected by different platforms.

Viime offers another value-added feature during data ingestion: imputation of missing data. Viime uses a sophisticated imputation strategy (Armitage, Godzien, Alonso-Herranz, López-Gonzálvez, & Barbas, 2015), heuristically classifying missing data as Missing Not At Random (MNAR) or Missing Completely At Random (MCAR). For MNAR data, the user can choose to replace the values with either zeros or half of the minimum value of that variable, while the MCAR options include imputation by Random Forest, K-Nearest Neighbors, the mean value, or the median value.

Finally, visualization of heatmaps, volcano plots, and network correlation diagrams, which all offer state-of-the-art web-based interactivity, can all be adjusted to include user selected subsets of data based on statistical significance or the particular interest of the investigator. This philosophy of interactivity will drive further development in viime as the platform expands its capabilities for further types of data analyses and visualization.

## Figures and Tables

**Figure 3:**
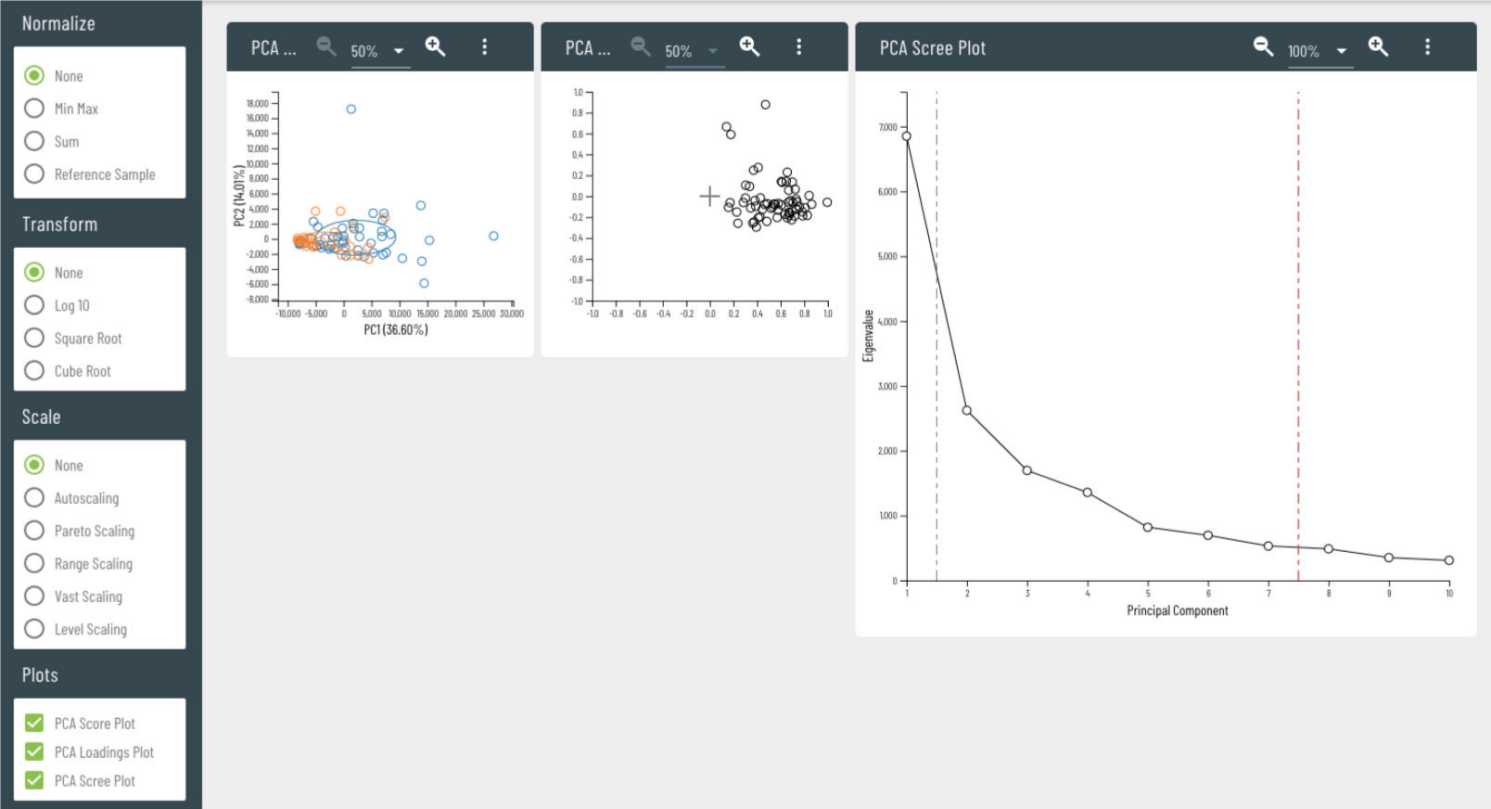
Dynamically updating customizable plots which animate to show immediate feedback when adjusting pretreatment options.

**Figure 5:**
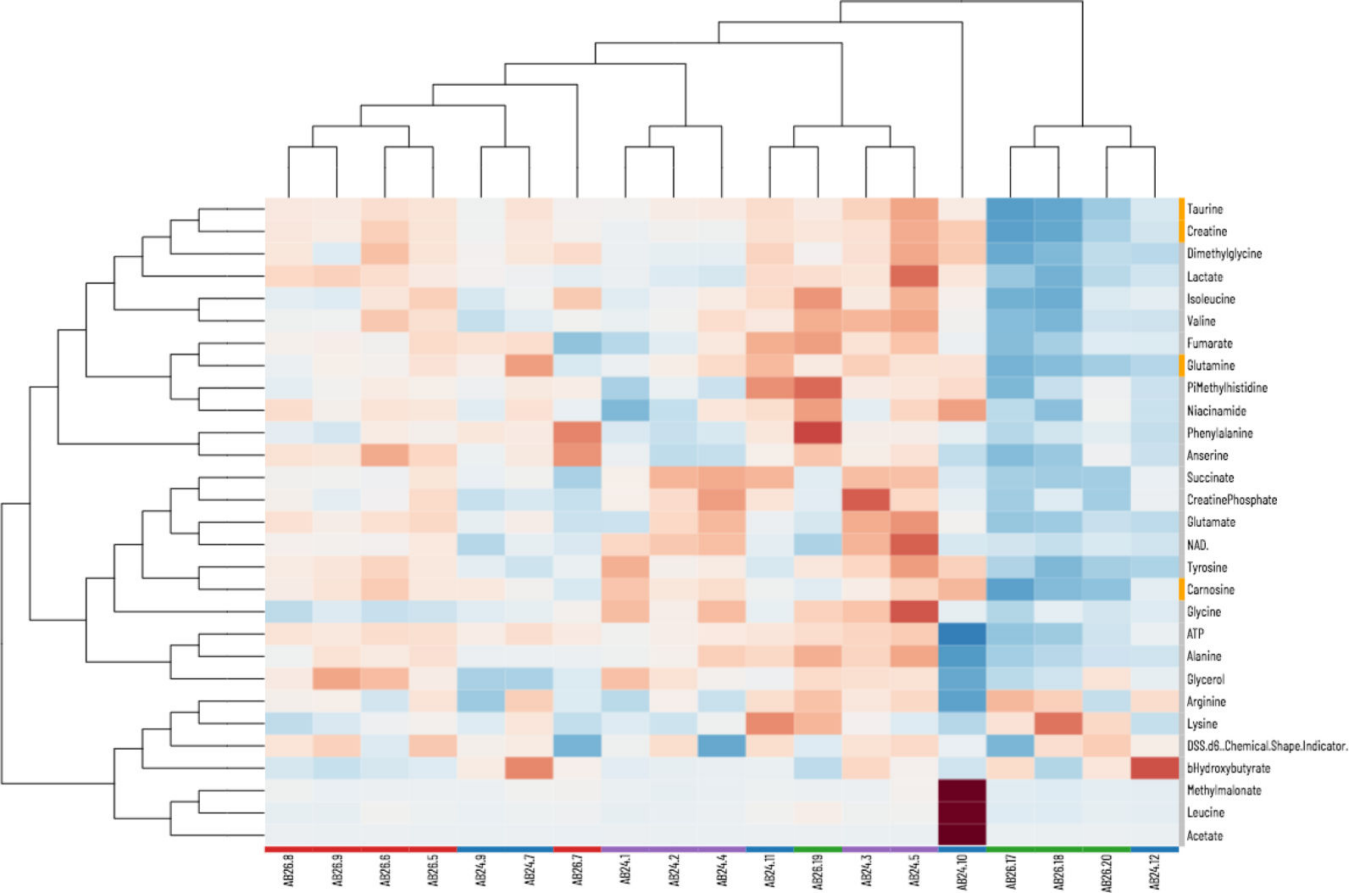
Heatmap with interactive collapsible clustering dendrograms for samples and metabolites.
